# Design of a Multifaceted Transitional Care Digital Intervention: Double Diamond Approach

**DOI:** 10.2196/99285

**Published:** 2026-11-12

**Authors:** Melissa T Baysari, Leslie E Oldfield, Melanie Bailey, Tin Fei Sim, Elizabeth Manias, Holly Foot, Faith R Yong, Mark Morgan, Claire Jackson, Ian A Scott, Laetitia Hattingh

**Affiliations:** 1 Digital Health Human Factors Group, Sydney Nursing School, Faculty of Medicine and Health, The University of Sydney, Level 8 Susan Wakil Health Building (A18), University of Sydney, New South Wales, 2006, Australia, 61 2 8627 9245; 2 School of Pharmacy and Pharmaceutical Sciences, Faculty of Health, Medicine and Behavioural Sciences, The University of Queensland, Brisbane, Queensland, Australia; 3 School of Diagnostic and Therapeutic Sciences, Curtin University, Perth, Western Australia, Australia; 4 Monash Nursing and Midwifery, Monash University, Melbourne, Victoria, Australia; 5 Rural Clinical School, Faculty of Health, Medicine and Behavioural Sciences, The University of Queensland, Brisbane, Queensland, Australia; 6 School of Public Health, Faculty of Health, University of Technology Sydney, Sydney, New South Wales, Australia; 7 Faculty of Health Sciences and Medicine, Bond University, Gold Coast, Queensland, Australia; 8 UQ/MRI Centre for Health System Reform and Integration, Brisbane, Queensland, Australia; 9 Queensland Digital Health Centre, The University of Queensland, Brisbane, Queensland, Australia; 10 Digital Health and Informatics Directorate, Metro South Health, Brisbane, Queensland, Australia; 11 Allied Health Research, Gold Coast Hospital, Gold Coast, Queensland, Australia

**Keywords:** medication, transition of care, discharge, digital intervention, design

## Abstract

**Background:**

Transitions of care, where patients move between clinical settings or providers, pose considerable risks to patient safety, situations exacerbated by the absence of adequate digital infrastructure that enables information exchange. The timeliness and accuracy of medication information transfer are central to ensuring patient safety during transitions of care, including post-discharge from the hospital. In designing a multifaceted digital intervention to support the transfer of medication information from hospital to community settings, it was necessary to gauge the views of all relevant stakeholders on the practical challenges involved in transferring medication information.

**Objective:**

This study aimed to define the challenges experienced by stakeholders, develop potential solutions, and use a collaborative approach to design a multifaceted digital intervention that spans hospital and community settings.

**Methods:**

This project involved multiple health service organizations in Queensland, Australia’s second-largest state geographically. Design of the intervention was guided by the United Kingdom Design Council’s Double Diamond Framework. This phased approach is particularly suitable for complex interventions involving multiple stakeholders. Over 12 months, we held an initial workshop with consumers (n=4), hospital clinicians (n=6), and primary care clinicians (n=7), and then convened over 50 hours of committee meetings, working groups, and consultations, undertook a system-walkthrough, and assessed the feasibility of the intervention. Participants were patients with relevant lived experience, clinicians, and those with technical, clinical governance, policy, research, and digital health implementation expertise.

**Results:**

Challenges described by workshop participants included fragmented digital systems across care settings, delays in discharge documentation reaching primary care clinicians, and current systems that function as incomplete or inconsistently used sources of medication information. Based on stakeholder consultation, our multifaceted intervention comprises both clinician- and patient-facing elements. These include a hospital-based Pharmacist Navigator role to facilitate post-discharge handover; a clinical dashboard for identifying and stratifying patient risk of medication misadventure; a software application for post-discharge pharmacist-led medication review and communication of the review to the patient’s general practitioner (GP); a Pharmacist Navigator notifying GPs of a patient’s hospital stay; patient education materials; and online training for community pharmacists conducting the medication reviews.

**Conclusions:**

Designing a multifaceted digital intervention to support medication information transfer at hospital discharge required significant time, effort, coordination, and engagement from a large number of stakeholders with various roles and areas of expertise. Key learnings are that transitional care interventions must be flexible and adaptable to align with the complex, evolving nature of health care, and that the success of a digital intervention relies heavily on human engagement and ongoing support.

## Introduction

Transitions of care, where patients move between clinical settings or providers, pose considerable risks to patient safety [1,2]. These risks gained worldwide attention in 2017 with the publication of the World Health Organization’s (WHO) *Third Global Patient Safety Challenge: Medication Without Harm*, where transitions of care featured as a priority area for action with a specific focus on medication continuity [3]. Hospital discharge is a particularly risky time, when errors in medication lists or summaries frequently occur, with one systematic review reporting that at least one in two patients experience an error or unintentional medication discrepancy at this transition point [4].

Errors in the transmission of medication information at hospital transitions arise from the complex interaction of multiple contributing factors [5,6]. A key factor is whether timely and accurate handover of medication information to community providers has occurred [7]. Delayed information transfer to providers and/or patients, and incomplete, inaccurate, or ambiguous information can result in medication omissions, duplications, and discrepancies. Interventions targeting errors at this transition point, therefore, focus on improving communication of medication information between patients and providers across care settings [6,7].

As digital systems become more commonplace in hospital and community settings, technology may offer a solution to streamline or even automate the transfer of medication information post-discharge. “Digital maturity” is considered to be critical for minimizing threats to patient safety at this transition point [8]. However, in Australia and many international health systems, the digital systems that underpin care delivery across settings are fragmented [9]. The disjointed nature of data flow across such systems is likely to exacerbate the post-discharge information transfer problem, making it challenging for providers and patients to know what information within which digital system is the most current, accurate, and accessible.

Medication reviews conducted in community settings serve as an important safeguard for identifying and reducing errors and discrepancies in medication information during transitions of care. In Australia, government-funded services available to patients include MedsChecks [10], in-pharmacy medication reviews performed by community pharmacists, and Home Medicines Reviews (HMRs) [11], which are in-home reviews performed by credentialed HMR pharmacists on referral from a general practitioner (GP). Despite the availability of these services, uptake of MedsChecks and HMRs following discharge from hospital is low [12,13].

Within this context of disconnected care, we embarked on designing and pilot testing a multifaceted digital intervention to support the transfer of medication information from hospital to community settings. In particular, to contribute to existing research on transitional care interventions, we aimed to define the challenges experienced by stakeholders, develop potential solutions, and use a collaborative approach to design a multifaceted digital intervention that spans health care settings.

## Methods

### Setting

This project involved multiple health service organizations in Queensland, Australia’s second-largest state by geographical size. The state’s population is relatively decentralized, with approximately half of its population residing outside the greater metropolitan area [14]. In Australia, responsibility for health care is shared between the federal, state, and territory governments, each performing different roles. The federal government bears responsibility for universal, nationwide funding and health policy (eg, Medicare), while state and territory governments manage the delivery of public hospital services and community health [15]. In Queensland, Hospital and Health Services (HHSs) are responsible for delivering public HHSs, while Primary Health Networks support primary care linkage and integration with secondary care. This shared responsibility is also reflected in the digital systems in place. State and territory governments are responsible for delivery of digital infrastructure at the state/territory level, such as hospital electronic medical records, while the federal government is responsible for delivery of national digital infrastructure, such as the My Health Record (MyHR), Australia’s nationwide consumer-controlled electronic health record [16].

Health information, including medication information, is sent from approved software (eg, hospital electronic medical records) to the MyHR using secure connections approved by the Australian Digital Health Agency, the national authority responsible for leading the national digital health strategy. The MyHR system uses HL7 Fast Healthcare Interoperability Resources (FHIR) as its modern data-sharing standard, transitioning alongside historical HL7 CDA (Clinical Document Architecture) frameworks. The MyHR may be securely accessed by consumers and clinicians in hospital and community settings to support care provision.

### Approach

The work described here is one component of a large trial to develop and evaluate a multifaceted intervention to optimize medication information handover after discharge from hospital (OPTMED-D [Optimizing Medicine Information Handover After Discharge]) [17]. Our process for designing the intervention was guided by the United Kingdom Design Council’s Double Diamond Framework [18]. This framework outlines 4 core processes for designing and delivering services, tools, and systems, as shown in Figure 1.

**Figure 1.**
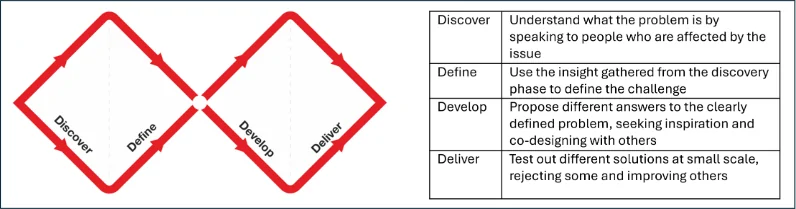
The double diamond framework and process [18,19] used to design the multifaceted digital intervention.

In phase 1 (*discover*), reported elsewhere [20], a series of 12 focus groups (n=75 participants) were held to understand the current challenges experienced by hospital and primary care clinicians and consumers with respect to medication information handover at discharge. Phase 2, described here, took learnings from the *discover* phase to *define, develop,* and *deliver* the intervention. Phase 3, yet to be completed, will evaluate our intervention in a large controlled trial [17]. Table 1 outlines the methods used in each phase of the double diamond approach, in sequence.

**Table 1.** Summary of methods, goals, and participants involved in each phase of the double diamond approach used to design the multifaceted intervention.

Phase	Discover^a^	Define	Develop	Deliver
Methods	Focus groups	Online workshop	Committees Work group meetings Consultations	Validation testing of clinical dashboard System walkthrough of medication management software Feasibility testing of digital components
Goals	To understand current challenges experienced by hospital and primary care clinicians and consumers with respect to medication information handover	To define the challenges and explore participants’ initial thoughts on a multifaceted intervention to support transfer of medication information	To discuss potential components of the proposed intervention with respect to need and feasibility	To operationalize each component of the multifaceted intervention, determine if it is fit for purpose, identify usability issues, and test the feasibility of each digital component
Participants	75 participants	17 participants	50 hours (approximate)	25 test patients 5 participants (feasibility testing)

a Detailed methods and findings are reported elsewhere [20].

### Defining the Challenges

To define the challenges and explore participants’ initial thoughts on a multifaceted intervention to support transfer of medication information, an online workshop was held with clinicians and consumers. To recruit participants for the workshop, all active participants from focus groups in phase 1 were invited. The workshop was facilitated by 5 experienced qualitative researchers (LH, LEO, MTB, EM, and HF) and ran for approximately 1.5 hours. Researchers initially presented a summary of the findings that emerged from the *discover* phase, and participants were then divided into smaller groups to discuss their thoughts on the key challenges related to the transfer of medication information and their ideas on potential solutions. Questions used to guide the workshop discussion appear in Section S1 in Multimedia Appendix 1.

The workshop, including discussions within each smaller group, was video-recorded with participant consent. The recordings were reviewed by a member of the research team (LEO) to complement detailed note-taking. At the conclusion of the workshop, each breakout group leader provided a verbal debrief, summarizing key discussion points.

Data were analyzed during a follow-up meeting with breakout group leaders. The meeting was conducted to review workshop notes and identify key themes from the *define* phase via a discussion and consensus approach, which were subsequently used to inform the *develop* phase of the process.

### Developing Solutions

Several committees and working groups were established at the time of project commencement, each with relevant expertise and local knowledge to inform intervention development (Table 2 and Figure 2). To recruit consumers to the Consumer Reference Group, expressions of interest were distributed to consumer organizations (ie, Health Consumers Queensland) and to Consumer Advisory Groups within health services. To be eligible to join the Consumer Reference Group, consumers needed to have experienced a hospital discharge in the last 12 months and be taking at least one medication.

**Table 2.** Project committees and working groups that informed multifaceted intervention development.

Group	Composition	Meeting frequency	Purpose	Development phase hours^a^
Steering Committee (n=15)	Project investigators (n=5), representatives from partner organizations (HHSs^b^, PHNs^c^) (n=9), and consumer (n=1)	Quarterly	To provide advice on OPTMED-D^d^ project design, implementation, and analysis	7
Consumer Reference Group (n=8)	Project investigators (n=2) and consumers (n=6)	Quarterly	To provide advice to the research team on the direction and approach of the OPTMED-D research project, including the multifaceted intervention; in particular, to provide advice on consumer-facing aspects (eg, training on the My Health Record, patient leaflets, etc)	3
Digital Health Committee (n=16)	Project investigators (n=4), consumer (n=1), and representatives from relevant partners with expertise in digital systems, including HHSs, PSA^e^, PHNs, AdPha^f^, government (Queensland Health) (n=11)	Bimonthly	To oversee development, implementation, and evaluation of the digital components of the intervention	4
Directors of Pharmacy Working Group (n=8)	Directors of pharmacy, or their delegate, from each participating hospital and health service	Monthly	To provide advice on aspects of the project and intervention that relate to hospital pharmacy services (eg, risk assessment process)	8

a Based on actual total duration of meetings.

b HHS: Hospital and Health Service.

c PHN: Primary Health Network.

d OPTMED-D: Optimizing Medicine Information Handover After Discharge project.

e PSA: Pharmaceutical Society of Australia.

f AdPha: Advanced Pharmacy Australia.

**Figure 2.**
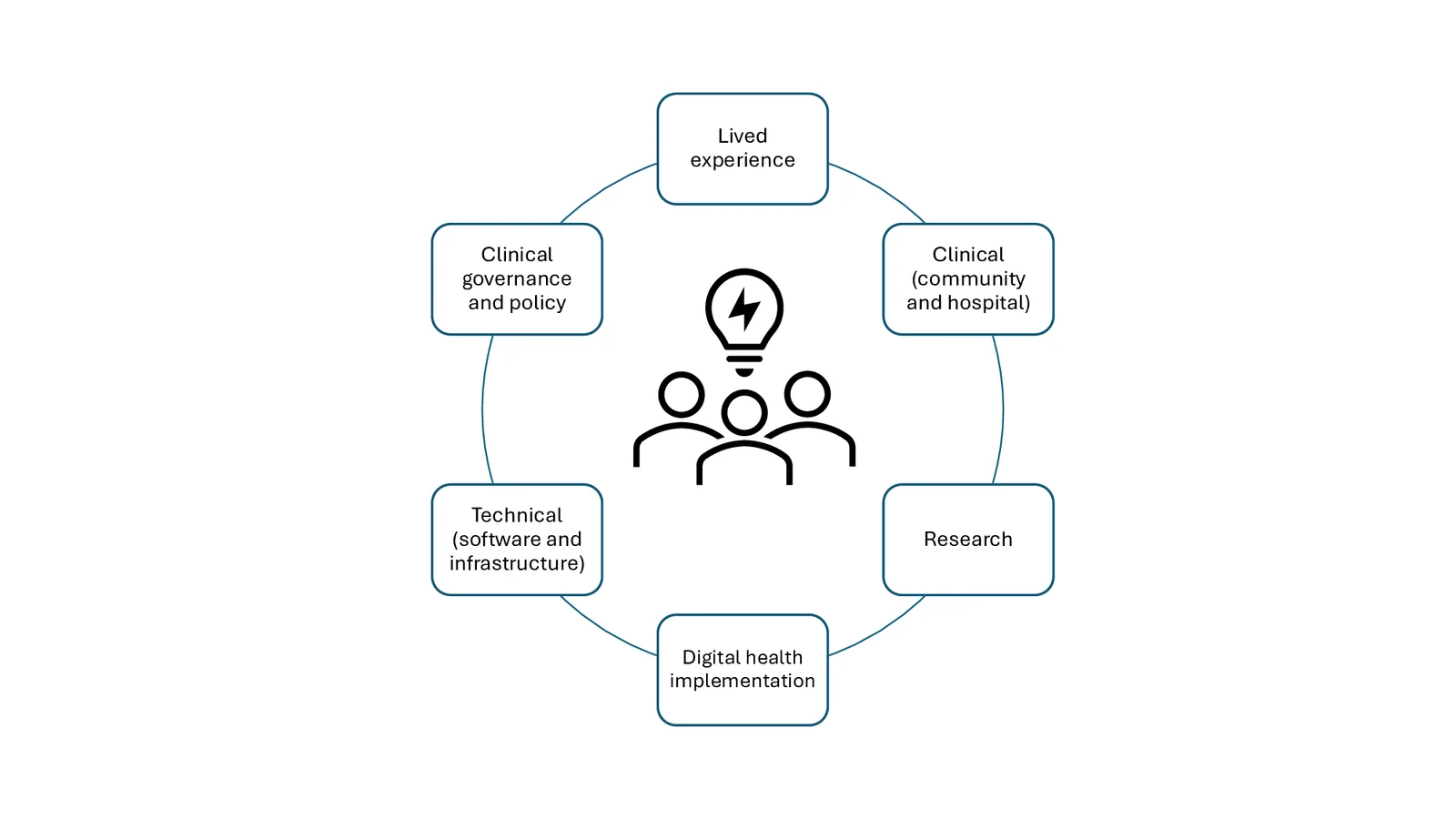
Role of individuals and groups involved in the design of the multifaceted intervention. Notes were taken by a member of the research team (LEO) during all regular and ad hoc stakeholder meetings (total >50 h). Summaries (ie, minutes) were prepared and distributed to participants, who confirmed that the summaries were accurate in capturing the meeting content.

Initially, findings from the *discover* and *define* phases were presented to each stakeholder group, and participants discussed potential components, both digital and nondigital, of a proposed multifaceted intervention (Figure 3) with respect to need and feasibility. In particular, feedback was sought from stakeholders on which digital tools and infrastructure were currently available to support the process outlined in Figure 3, the scope of the intervention, and guidance on current contextual and environmental factors likely to impact the development, implementation, and ultimate success of the intervention.

**Figure 3.**
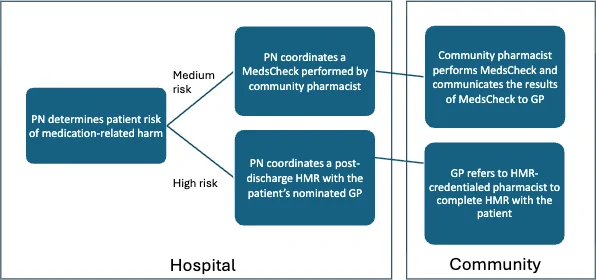
Proposed components of the multifaceted intervention. Boxes indicate the settings where components were operationalized (ie, hospital or community settings). GP: general practitioner; HMR: Home Medicines Review; PN: Pharmacist Navigator.

In addition to the regular meetings (Table 2), a large number of consultations were held with other key individuals and groups to scope the current digital landscape, infrastructure, workflows, and systems in order to determine what intervention components would be feasible (Figure 3). Participants also discussed previously trialed or tested interventions to explore learnings from these experiences (ie, what worked and what did not work). Meetings were held both for information gathering and decision-making with representatives from the Australian Digital Health Agency, responsible for Australia’s national digital health strategy, the state department of health (ie, Queensland Health [QLD Health]), and local hospital informatics teams.

For data analysis, several working groups, comprising study investigators and researchers, were established to refine ideas and troubleshoot issues, including the Primary Care Working Group, the Audit Working Group, and the Digital Working Group. No formal thematic analysis of meeting data was performed; however, flowcharts and standard operating procedures (SOPs) were created for each stakeholder group, which outlined the multifaceted intervention and how it should be adopted in practice. Feedback was sought from all relevant stakeholders (Table 2) on the flowcharts and SOPs, and they were modified to improve clarity and understandability for end users.

### Delivering a Solution

Table 3 outlines the digital components of the intervention that were identified in the *develop* phase to support the process outlined in Figure 3, and describes how each component would be operationalized during the deliver stage (ie, tested and returned to the *develop* phase when refinement was needed).

**Table 3.** Digital components of the multifaceted intervention and the “deliver” approach.

Digital tool/system/process	Target end user	Purpose	Deliver (testing) approach
Clinical dashboard with risk classification tool^a^	Pharmacist navigator	Identify patients residing in relevant study postcodes, assist with determining each patient’s risk of experiencing medication-related harm and each patient’s path for post-discharge handover and monitoring	Validation testing
My Health Record	Patient, GP^b^, and community pharmacist	Act as a source of medication-related information, including a patient’s DMR^c^	Feasibility testing
Medication management software^a^	Community pharmacy owner/manager, pharmacist navigator, and community pharmacist	Book a post-discharge MedsCheck appointment, conduct a MedsCheck, and communicate results of the MedsCheck to the GP	System walkthrough and feasibility testing
Online training^a^	Community pharmacist	Educate pharmacists on transitions of care, and train on digital systems and intervention processes	Validation testing

a Indicates that the tool/system/process was developed or modified for the purposes of our study.

b GP: general practitioner.

c DMR: discharge medication record.

#### Validation Testing of the Clinical Dashboard

Research team members tested the new clinical dashboard for data accuracy and ease of use by searching for individual patients, reviewing a sample of patients (n=25), and cross-checking the dashboard outputs against manual calculations of each patient’s risk level.

#### System Walkthrough of Medication Management Software

Three researchers with expertise in usability evaluation (MTB), hospital pharmacy (LH), and community pharmacy (MB) independently walked through the medication management software for organizing and reporting a post-discharge MedsCheck to determine whether it was fit for purpose and to identify any usability issues. Usability issues identified by each researcher were compiled into a master list, and the researchers came together to collectively review each issue and assign a level of severity to each problem based on a well-established severity rating scale for usability problems (1=cosmetic problem, 2=minor usability problem, 3=major usability problem, 4=usability catastrophe) [21]. Usability problems were communicated to the vendor, who modified the medication management software to address all usability issues with a severity score of 3 or 4.

#### Feasibility Testing for All Digital Components

In undertaking preliminary testing of the feasibility of digital components of the multifaceted intervention, relevant end users completed predefined tasks required as part of the multifaceted intervention using simulated patient data while being observed by a researcher (Table 4). This initial testing was performed with one consumer, who was a member of the Consumer Reference Group, 2 community pharmacists (one internal and one external to the research team), and 2 GPs (one internal and one external to the research team).

**Table 4.** Tasks tested by each end user group during feasibility testing of the multifaceted intervention.

End user	Task
Community pharmacist	Complete and document the results of a post-discharge medication review (MedsCheck) in the medication management software
Community pharmacist	Communicate the results of a MedsCheck to a general practitioner
General practitioner	Receive and review the results of a MedsCheck
Consumer	Review medication information on the My Health Record

A member of the research team with expertise in usability and health technology evaluation took handwritten notes during each session and recorded how and whether each task was completed successfully, as well as any issues or problems encountered by the user. Following testing, the researcher conducted a short debriefing session with participants to explore the feasibility of the digital components (see Section S2 in Multimedia Appendix 1). Interview questions explored the 6 relevant areas of focus in feasibility studies: acceptability, demand, implementation, practicality, adaptation, and integration [22].

During data analysis, handwritten interview notes, together with observation notes, were subsequently reviewed and discussed with a clinician member of the research team to establish consensus on challenges and issues associated with each component of the multifaceted intervention.

### Ethical Considerations

This study received ethics approval from the Gold Coast Health Human Research Ethics Committee (HREC/2023/QGC/101063) in November 2023. All participants gave written informed consent prior to participating. Primary care clinicians were compensated for their time in accordance with QLD Health policy and local Primary Health Network recommendations. Consumers were compensated for their time at a rate of AUD $45 (US $31.40) per hour. All data were deidentified prior to analysis.

## Results

### Defining the Challenges in Medication Information Transfer

In total, 17 participants took part in the workshop held in October 2024. These included 4 consumers, 3 hospital pharmacists, 4 community pharmacists, 1 GP pharmacist, 2 hospital doctors, 1 hospital nurse navigator, and 2 GPs.

Challenges described by participants were highly consistent with those identified in our initial *discover* phase [20] and included fragmented digital systems across care settings, delays in discharge documentation reaching primary care clinicians, and MyHR functioning as an incomplete or inconsistently used source of medication information. Participants expressed uncertainty regarding whether a new digital platform to support medication communication was needed, and there were mixed views on whether existing infrastructure, such as MyHR, could be adapted or replaced. Participants highlighted variability in patients’ access to and familiarity with digital technologies.

With respect to designing potential solutions, participants emphasized the importance of establishing a shared, reliable “source of truth” for medication information. Some participants supported strengthening MyHR to fulfill this role, contingent on access for community and credentialed pharmacists and the ability to upload reports from medication reviews (MedsChecks and HMRs). Others supported the development of a simplified, medication-focused platform, provided it could integrate with existing systems and minimize manual data entry. Participants also wanted automated hospital admission and discharge notifications to be sent to the appropriate community clinicians (GPs and community pharmacists) to inform them that their patient was in hospital, and to prompt review of the patient’s MyHR. Participants stressed the importance of continuing to provide a paper-based, patient-friendly medication list at discharge, given the wide variability in consumers’ access to and comfort with digital technologies.

Participants supported the idea of a new hospital-based role (ie, “Pharmacist Navigator”) to support continuity of care post-discharge by liaising with GPs, community pharmacists, and allied health providers. They also suggested that a Pharmacist Navigator could follow up either with primary care clinicians (ie, GPs and pharmacists) or with patients post-discharge to check whether a medication review had been completed as planned. Despite the overall positive reactions, some participants questioned how Pharmacist Navigators would work alongside existing hospital pharmacy teams and in which contexts this new role would function (eg, after-hours coverage and private hospital settings).

### Formulating the Components of the Multifaceted Intervention

#### Intervention Components

Findings from the workshop and stakeholder consultations were used to formulate the components of our multifaceted intervention, which comprised clinician and patient-facing elements, as well as digital and nondigital components, described below. An example flowchart and SOP for one end user group (community pharmacists) appear in Multimedia Appendix 2.

While the proposed intervention purposely leveraged existing digital systems, infrastructure, and processes, the findings specifically informed the development of a new clinical dashboard for patient identification and risk stratification of medication-related harm to be used by hospital-based Pharmacist Navigators. Findings were also used to identify necessary modifications to an existing medication management software application routinely used by community pharmacists. This application is currently used in approximately 90% of Australian pharmacies to book, perform, and document extended health services (eg, vaccinations). Needs and preferences for revising the medication management software were then communicated to the vendor, who developed an initial prototype of the software for researcher testing.

#### Hospital Pharmacist Navigators

The multifaceted intervention commences in hospitals, with a dedicated resource: a Pharmacist Navigator, who participates in discharge planning and determines each patient’s risk of medication-related hospital readmission through a risk-stratification process using the clinical dashboard (described in the next section).

After identifying a patient’s risk level, Pharmacist Navigators coordinate post-discharge handover and monitoring (Figure 3). If a patient is deemed to be at medium risk of medication-related harm (pathway 1), the Pharmacist Navigator coordinates a post-discharge medication review (post-discharge MedsCheck) with the patient’s community pharmacist. An online booking system is available in most cases to enable the timely scheduling of a MedsCheck. If a patient is deemed to be at high risk of medication-related harm (pathway 2), the Pharmacist Navigator coordinates a post-discharge HMR referral to the patient’s nominated GP and notifies the GP via telephone.

For pathways 1 and 2, Pharmacist Navigators generate and send a letter to the patient’s GP informing the GP of the patient’s recent hospital admission and the prescribed post-discharge MedsCheck or HMR. This letter automatically results in the generation of text in the relevant hospital electronic medical record indicating that the GP has been contacted by the team.

#### Clinical Dashboard

To support Pharmacist Navigators in identifying patients residing in postcodes serviced by community pharmacists participating in the study, and to determine which path a patient will be assigned to post-discharge based on risk level, the research team worked with state clinical informatics teams to develop a clinical dashboard with an embedded clinical risk prediction tool, the PRIME score. PRIME is a rules-based algorithm using 8 variables that outputs a low (0%‐14.9%), moderate (15%‐24.9%), and high (≥25%) risk score for medication-related harm and hospital readmission following hospital discharge [23]. This novel, state-owned and operated dashboard is integrated with the hospital electronic medical record and displays each patient’s PRIME score calculated using relevant data, and also presents key patient demographics (eg, postcode) and clinical (eg, renal function) information for the study and to facilitate pharmacy review (Figure 4). Access to the electronic medical record is under QLD Health policy and provisioned under role-based needs. The clinical dashboard is secured on the QLD Health network, with access granted upon request to QLD Health staff who have access to the electronic medical record. All clinical systems, databases, and dashboards reusing electronic medical record data are governed by appropriate technical and clinical governance structures, as required by QLD Health, and by state and national legislation.

**Figure 4.**
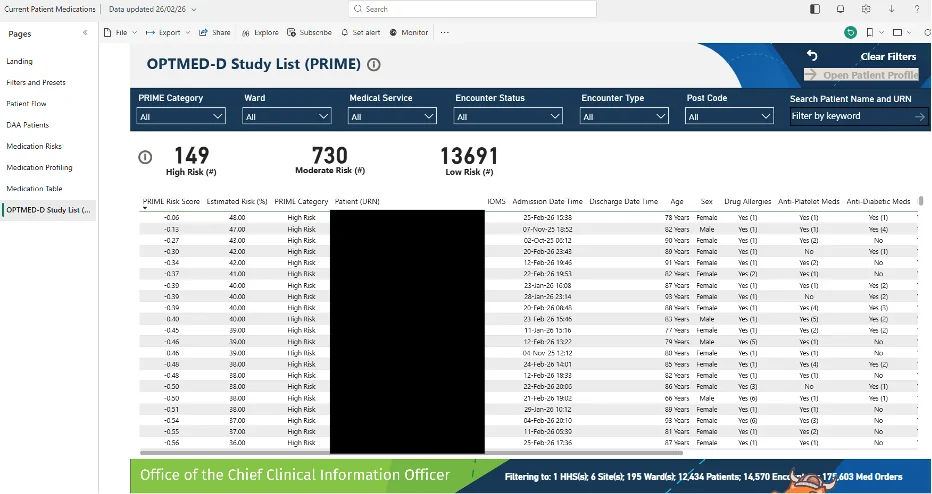
Screenshot of a clinical dashboard that allows Pharmacist Navigators to identify patients residing in postcodes and to view each patient’s PRIME score.

#### Community Pharmacists

Community pharmacists, once onboarded to the study, receive online training on transitions of care, post-discharge MedsChecks, and the modified medication management software. The software is ISO-27001-certified, and its security approach is aligned with Australian Government security expectations. They conduct a patient’s post-discharge MedsCheck using the software and then electronically send the results of the MedsCheck (ie, a post-discharge MedsCheck report) to the patient’s nominated GP via secure messaging, which includes email or SMS. This messaging functionality ensures that patient and user data are encrypted in transit and at rest, stored on secure servers in Australia, and designed to be accessible only to intended users throughout the information flow.

#### GPs

GPs receive information about how the multifaceted intervention operates. For their patients who are deemed high risk by Pharmacist Navigators (pathway 2), GPs receive a letter informing them of their patient’s recent hospital admissions and the recommendation for a post-discharge HMR. They then coordinate a referral for an HMR and follow SOPs for booking an HMR with a credentialed pharmacist. For patients who are deemed medium risk by Pharmacist Navigators (pathway 1), GPs receive a letter informing them of their patient’s recent hospital admissions and the arranged post-discharge MedsCheck, and subsequently receive the report from their patients’ post-discharge MedsCheck electronically via the medication management software’s secure messaging functionality (email or SMS).

#### Patients

During the discharge process, patients receive a patient-friendly discharge medication record, a brochure, and education on MyHR, including how to access the system and view medication information. This easy-to-read brochure was developed with the assistance of the consumer reference group.

#### Training

A series of online training materials was developed for community pharmacists, and study information was also disseminated to credentialed HMR pharmacists and GPs. Community pharmacist training on transitions of care was developed in partnership with the Pharmaceutical Society of Australia, and the medication management software instructions were developed in partnership with the software vendor. Community pharmacist training (approximately 2 h) comprised an online interactive module that addressed the role and responsibilities of community pharmacists during transitions of care, structured medication reconciliation, identification and prioritization of patients at risk of medication-related harm, and the components of pharmacist-led, person-centered transitions-of-care services. Completion of the training would not be mandatory for participation in the intervention; however, it was intended to support pharmacists’ understanding of transitions of care and provide practical, evidence-based strategies for delivering the intervention in the subsequent trial, while also contributing to their continuing professional development requirement.

### System Walkthroughs of Medication Management Software

Walkthroughs of the medication management software took place in August 2025. Researchers identified 15 usability problems with the tool; 6 were deemed minor usability issues, 4 were rated as major issues, and 5 as catastrophic issues, requiring the system to be modified before use. Table 5 shows all catastrophic issues and researcher recommendations made to, and addressed by, the medication management software vendor.

**Table 5.** Catastrophic usability issues identified by researchers during walkthroughs of the medication management software.

Issue	Recommendation
Indicating whether a patient has an allergy is mandatory, but specifying what the patient is allergic to or the reaction experienced is nonmandatory	Make it mandatory for users to specify the allergy type and reaction
Clicking “Other (please specify)” for the issue and outcome results in these fields being blank in the MedsCheck report, even if these issues and outcomes are entered into the Notes box	If the user clicks “Other (please specify),” a separate box should appear for data entry, and this data should appear in the MedsCheck report
In the outcomes list, the verb tense of “Reviewed by GP^a^” is incorrect, making it confusing for users	Change verb tense to “Review by GP” to make clear to users that this still needs to take place
When additional text is entered into the MedsCheck introductory letter (eg, recommendations), this is not pulled across into the introductory text that appears in the MedsCheck report	All additions to the letter need to appear in the MedsCheck report letter
GP report does not include patient allergies or underlying conditions	GP report should include patient allergies, underlying conditions, and any other data that has been entered in the MedsCheck form

a GP: general practitioner.

### Preliminary Testing of the Feasibility of the Multifaceted Intervention

No major problems were identified; however, several minor issues were uncovered during testing, requiring some small modifications to the intervention, including the instructions and information provided to stakeholders about the digital components of the intervention. These issues and solutions are listed in Section S3 in Multimedia Appendix 1.

During testing with community pharmacists, it became apparent that some steps in the instructions on how to complete a post-discharge MedsCheck were missing and could have been clearer in some areas. Testing with GPs demonstrated that GPs and their practice managers might view study communications (MedsCheck reports and letters) as unsolicited and irrelevant (ie, spam) and subsequently delete them.

Testing of the clinical dashboard revealed that the tool was easy to use, particularly for patient identification. However, the team identified some inaccuracies with the PRIME score, which, on further testing and validation, were due to incomplete electronic medical record data used to calculate the score. In particular, the “living alone” field was inconsistently documented by clinicians in practice, as there was no specific field in the electronic medical record for this. To ensure a consistent approach in risk stratification for the next phase of the project (ie, randomized controlled trial), Pharmacist Navigators will be required to manually add the “living alone” variable to each patient’s PRIME score calculation before assigning a patient to a post-discharge pathway.

## Discussion

### Principal Findings

This study is one of the first to describe step-by-step the processes used to design a multifaceted intervention aimed at improving post-discharge transfer of medication information to community providers. The study showed that the processes of gathering information on the context and challenges and determining the components of the intervention were not straightforward and took over a year. It required more than 50 hours of consultation with stakeholders whose expertise ranged from lived experience to technical knowledge. Feasibility testing within hospital and community settings proved extremely valuable for identifying issues and pain points likely to negatively affect the effectiveness of the intervention when implemented. The outcome of the double diamond approach was a multifaceted intervention that included digital components in both hospital and community settings, relying heavily on human involvement.

Although slightly nuanced, the challenges and goals uncovered in the *define* phase of our study were highly consistent across all participant groups. Stakeholders recognized the importance of timely and accurate handover of medication information post-discharge and all reported issues related to fragmented systems that impact the continuity of care. In contrast, the *develop* phase, where we set out to identify feasible and practical solutions to the challenges uncovered, proved to be complex and difficult.

Understanding contextual factors that would support or hinder intervention success was particularly challenging for the digital components of the intervention, as the digital health landscape, including systems and infrastructure, is not static and, in some cases, rapidly changing [24]. For example, over the course of our consultations, we learned that the MyHR functionality for pharmacists was due to change (where uploading of MedsCheck and HMR reports to the system would be available), possibly during the final phase of our project, which is a cluster randomized controlled trial [17]. Thus, developing an intervention that is agile and modifiable to align with the complex adaptive nature of healthcare is likely to be more effective and sustainable than a rigid, static intervention. Without the careful and comprehensive preparation for study implementation, as detailed in this paper, optimal adoption across the broader health system is unlikely.

In contrast to designing an intervention within a single, circumscribed setting, designing a multifaceted intervention that crosses care settings requires multiple interconnected components that address contextual challenges and meet the needs of each stakeholder group. Consistent with previous work on multidisciplinary transitional care interventions [25,26], our intervention required coordination, flexibility, and accommodation of various clinical responsibilities, and adaptation to variability in communication systems, workflows, and patient needs.

We knew at the outset of our project that a single-element intervention would not suffice and that a multifaceted intervention would be needed [27]. Although we set out to design a primarily digital intervention, it quickly became apparent that this would need to be heavily supplemented by complementary strategies, including consumer and clinician education and human resources (ie, a hospital-based pharmacist navigator to initiate the information transfer process). Our validation testing identified data quality issues related to the use of the PRIME score and, like other research [28,29], highlighted challenges and risks associated with decision support tools relying on incomplete data, necessitating human or manual support. Implementation science frameworks, such as the Consolidated Framework for Implementation Research [30] and the nonadoption, abandonment, scale-up, spread, and sustainability (NASSS) framework [31], point to implementation strategies surrounding digital tools as crucial determinants of uptake and long-term adoption. Recognizing these strategies as core elements of our multifaceted intervention, we deliberately designed and tested them alongside the digital components.

The multifaceted clinician-driven nature of our intervention is in line with findings from a rapid review of interventions to facilitate safer medication management at transitions of care, which found that the most effective strategies were those led by pharmacists and delivered in collaboration with other health care providers [32]. A similar review that focused on digital interventions highlighted that digital approaches are unlikely to minimize information errors at transition points without active engagement from all key stakeholders, including patients/carers, and both hospital and community providers [33]. Our study reinforced these findings and clearly demonstrates that digital interventions alone are insufficient; they must be complemented by other elements to be successful.

### Strengths and Limitations

This study outlines, in depth, the double diamond approach we used to design a transitional care intervention. However, it did not examine the uptake and impacts of the intervention once implemented, which will be undertaken in the next phase of our planned research. It also did not evaluate the feasibility and cost of maintaining all the intervention components, potentially limiting their continued use following the trial. Although we engaged a large and diverse group of stakeholders, including consumers and both community and hospital clinicians, all participants were self-selected and, therefore, the views presented may not reflect those of the broader stakeholder population. Feasibility testing involved only 5 participants, which limits the generalizability of the conclusions drawn.

With respect to implications for practice and the scale-up of a multifaceted intervention such as this, as previously mentioned, the majority of health policies relating to transitions of care and medication reviews in Australia are federally funded, while state/territory hospitals are funded by their respective jurisdictions. This makes scaling up a transitional care intervention that crosses state/territory and federal boundaries highly challenging. The use of different clinical software in hospitals, pharmacies, and general practices also acts as a barrier to scaling up, indicating that significant further investment is likely needed for broader implementation and testing. Similarly, the pharmacist navigator role is a crucial component of the multifaceted intervention and is likely to be a necessary condition for intervention success in reducing medication-related readmissions. Sustainability of this pharmacist role will require long-term resourcing and some thought about its positioning and value within broader hospital pharmacy teams.

### Conclusions

Designing a multifaceted digital intervention to support medication information transfer at hospital discharge required significant time, effort, coordination, and engagement from a large number of stakeholders with various roles and expertise. This project shows that effective transitional care interventions must be agile and modifiable to suit the complex and adaptive nature of health care environments, and that the success of a digital intervention relies heavily on human engagement and support.

## Supplementary material

10.2196/99285 Multimedia Appendix 1 Workshop discussion questions, feasibility testing questions, and findings and resulting changes from feasibility testing.

10.2196/99285 Multimedia Appendix 2 Flowchart and standard operating procedures for general practitioners.

## Abbreviations

AdPha: Advanced Pharmacy Australia
FHIR: Fast Healthcare Interoperability Resources
GP: general practitioner
HHS: Hospital and Health Service
HL7 CDA: Health Level Seven Clinical Document Architecture
HMR: Home Medicines Review
MyHR: My Health Record
NASSS: nonadoption, abandonment, scale-up, spread, and sustainability
OPTMED-D: Optimizing Medicine Information Handover After Discharge
SOP: standard operating procedure
WHO: World Health Organization
