# Supplementary material for: Design of a Multifaceted Transitional Care Digital Intervention: Double Diamond Approach
Source: JMIR Form Res. 2026 Nov 12;10:e99285. doi: 10.2196/99285 (PMC13759177; doi:10.2196/99285)
Supplement: Supplementary file 1 [file formative-v10-e99285-s002.pdf]

General Practitioner: OPTMED-D Trial Overview

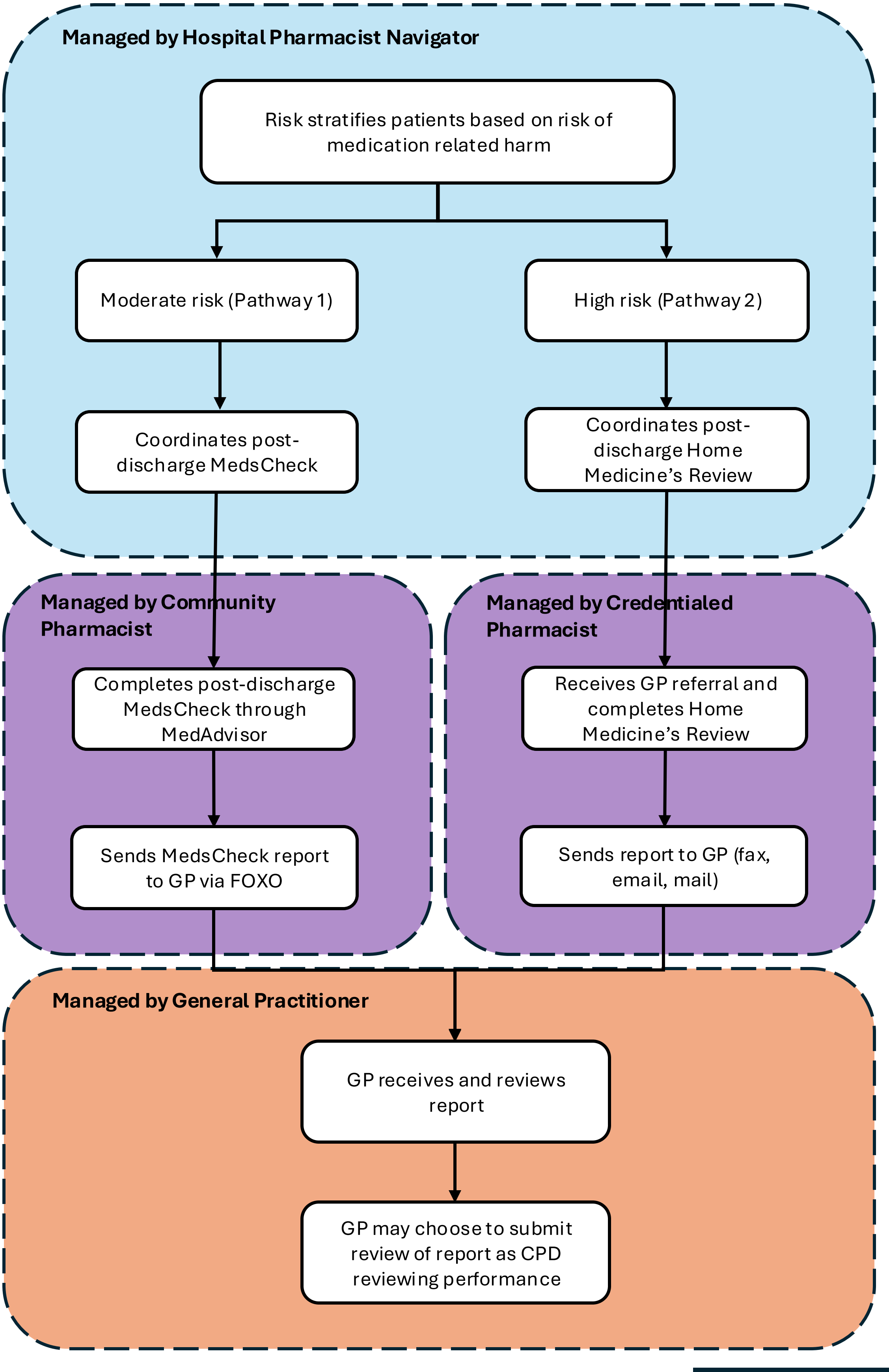

Contact:
OPTMED-D@uq.edu.au

What is OPTMED-D and what do I have to do to benefit my patients?

This document outlines the steps a General Practitioner (GP) may be involved in as part of the OPTMED-D study, depending on whether their patient is assessed as moderate- or high-risk for medication-related harm.

Step 1: Prior to discharge (Hospital-based process)

- Hospital Pharmacist Navigator conducts risk stratification to identify patients at moderate or high risk for medication-related harm.
- After patient consent:
  - Pathway 1 (Moderate risk): Pharmacist Navigator books a post-discharge MedsCheck at the patient's preferred community pharmacy.
  - Pathway 2 (High risk):
    - The Pharmacist Navigator contacts the patient's GP to coordinate a post-discharge consultation, where the GP can review the patient and coordinate an HMR referral.
    - The Pharmacist Navigator supports the GP to coordinate the HMR with a local Credentialed Pharmacist.

GP actions (high-risk only):

- Provide an HMR referral for the patient during the post-discharge consultation.
  - Referral can be completed using the practice's standard process (e.g., MedicalDirector template, secure messaging to the Credentialed Pharmacist, fax, or email).
  - If you do not have an established relationship with a Credentialed Pharmacist, the Pharmacist Navigator can assist in connecting you to a local Credentialed Pharmacist.

Step 2: At discharge (Hospital-based process)

No GP action required at this stage

- The Pharmacist Navigator uploads the Discharge Medication Record (DMR) to My Health Record.
- A physical copy of the DMR is also provided to the patient.

Step 3: Post-discharge

A. <u>Moderate-risk patients</u>

Community pharmacy actions:

- The patient's preferred Community Pharmacist conducts a MedsCheck using MedAdvisor and documents the result.
- Results are sent to the GP through MedAdvisor via FOXO - this will be in the form of an email to the Practice or GP or via SMS .

GP actions:

- GP or GP Practice will receive an email within their inbox (look out for an email with subject OPTMED-D).
- If this is the first time receiving a FOXO notification, the GP/Practice will be required to request a passcode and enter the passcode to access the MedsCheck result. This is only required on the first instance.
    - Detailed instructions are attached (document titled “MedAdvisor- Secure Comms”).
- Review and action the MedsCheck findings as clinically appropriate (e.g., changing medications, follow-up planning).

B. High-risk patients

Credentialed Pharmacist actions:

- Conduct the HMR post-discharge.
- Send the HMR report to the GP using the GP’s preferred communication method (e.g., secure messaging, fax, email, mail).

GP actions:

- Review and action the HMR report, according to Medicare requirements. This involves:
    - Discussing the results with the credentialed pharmacist
    - Developing a written medication management plan
    - Providing the written medication management plan to the patent’s community pharmacy

Step 4: Optional CPD

- Reviewing MedsCheck or HMR reports may be counted as a “reviewing performance” CPD activity.
- Suggested reflection questions are available on our website.

Contact Information

OPTMED-D Study Email: OPTMED-D@uq.edu.au
Principal Investigator: Laetitia Hattingh (laetitia.hattingh@health.qld.gov.au)
