# Supplementary material for: Design of a Multifaceted Transitional Care Digital Intervention: Double Diamond Approach
Source: JMIR Form Res. 2026 Nov 12;10:e99285. doi: 10.2196/99285 (PMC13759177; doi:10.2196/99285)
Supplement: Supplementary file 2 [file formative-v10-e99285-s001.docx]

**Multimedia Appendix 1**

**S1. Questions used to guide workshop discussions**

Breakout Room #1: Pharmacist Navigator

Role description, as outlined in Phase 1:

1. Assist with discharge planning and help the patient through the discharge process
2. Determine patient’s risk of readmission (they will stratify patients based on their readmission risk)
3. Coordinate post-discharge handover and prompt medication management review services, such as MedsCheck
4. Patients will be able to contact the Pharmacist Navigator post-discharge if they have any questions

Questions discussed:

1. Are there any other tasks the Pharmacist Navigator can or should do?
2. How can the Pharmacist Navigator integrate into the existing hospital teams?

Breakout Room #2: Post-discharge medication management review services

How it would work, as outlined in Phase 1:

- Pharmacist Navigator will liaise with community pharmacist to coordinate a MedsCheck for moderate-risk patients or use an App to make the MedsCheck appointment
- Pharmacist Navigator will liaise with credentialed pharmacist and GP to coordinate a Home Medicines Review (HMR) for high-risk patients
- The outcome of these MMR services will be communicated to the GP via a digital intervention

Questions discussed:

1. Practically, how should these results be communicated to the GP?
   a. How can these results be integrated into the GPs medicine record system?
2. How should we determine who is moderate-risk and who is high-risk?

Breakout Room #3: Digital intervention- communication between health professionals

Questions discussed:

1. How can we get existing systems to communicate with each other? What systems need to communicate?
2. How should GPs be informed of MedsCheck and Home Medicine Review outcomes?
3. How can we enable bidirectional communication?
4. Would a stand-alone system (app, website) work?

Breakout Room #4: Digital intervention- consumer information and communication

Ideas suggested in Phase 1:

1. Education campaign to inform consumers about post-discharge medication management review services and discharge medicine lists
2. Uncouple patient friendly discharge medicines list from the discharge summary
3. QR code in hospital to prompt questions to ask clinicians about medicines
4. Patient friendly discharge medicines texted to patients
5. QR code or App for patients at discharge to schedule MedsCheck or HMR based on their risk profile

Questions discussed:

1. How should we prioritise the ideas suggested?
   a. Which ones should we focus our attention on?

**S2. Questions used to guide discussion with end-users following feasibility testing**

- Was the multi-faceted intervention suitable and satisfying to use? Why? If not why?
- Was the multi-faceted intervention practical to use or deliver? Why? If not why?
- Did you have all the necessary resources to implement and use the intervention? If no, what else did you need?
- Was it possible to integrate the multi-faceted intervention into your existing ways of working? Any challenges with this?
- What did you like best about the multi-faceted intervention? Why?
- What did you like least about the multi-faceted intervention? Why?
- Is there any aspect, function or feature you would change or improve?
- What problems in need fixing before this intervention can be implemented in practice?

**S3. Findings and changes made as a result of feasibility testing**

| **Issue** | **Changes made to intervention** |
| --- | --- |
| When setting up the booking process, pharmacists are required to enter a booking appointment length for post discharge MedChecks, but no instructions are provided about this | In the instructions for setting up a booking Calander, specify that post-discharge MedsCheck appointments should be set to 30 minutes in length |
| When completing a MedsCheck, there is no option for ‘post-discharge MedsCheck’ on the drop-down list for reason for the MedsCheck | In the instructions for completing a MedsCheck, specify that pharmacists should click on ‘Significant medical event’ (i.e. the hospital visit) as the reason for the MedsCheck |
| When completing a MedsCheck, pharmacists need to select how they would like to communicate the MedsCheck report to GPs, but no instructions are provided about this | In the instructions for completing a MedsCheck, specify that pharmacists should select ‘Referral via secure message’ as their mode of communication |
| When completing a MedsCheck, before the MedsCheck report is sent to GPs, pharmacists see a message template that will accompany their MedsCheck report. It is not clear from the instructions that this template will pre-populate with information from the MedsCheck report (i.e. it should not be manually completed) | In the instructions for completing a MedsCheck, clearly specify that the fields in brackets in the message template will automatically populate, no manual data entry is required |
| GP practices are unaware of the study, perceive emails from the medication management application to be spam and delete the emails received containing MedsCheck results | In the information distributed to GPs, specify that GPs/GP practices will be sent an email from FOXO containing a link to a MedsCheck report.  Ensure that the study name is clearly visible in the subject line of emails |
